# From single gene analysis to single cell profiling: a new era for precision medicine

**DOI:** 10.1186/s13046-020-01549-3

**Published:** 2020-03-05

**Authors:** Maria Teresa Di Martino, Stefania Meschini, Katia Scotlandi, Chiara Riganti, Enrico De Smaele, Francesca Zazzeroni, Massimo Donadelli, Carlo Leonetti, Michele Caraglia

**Affiliations:** 1 Department of Experimental and Clinical Medicine, University of Catanzaro “Magna Graecia”, Catanzaro, Italy; 2 National Center for Drug Research and Evaluation, National Institute of Health, Rome, Italy; 3 IRCCS Istituto Ortopedico Rizzoli, Experimental Oncology Lab, Bologna, Italy; 4 Department of Oncology, University of Torino, Turin, Italy; 5 Department of Experimental Medicine, Sapienza University of Rome, Rome, Italy; 6 Department of Biotechnological and Applied Clinical Sciences, University of L’Aquila, L’Aquila, Italy; 7 Department of Neurosciences, Biomedicine and Movement Sciences, Section of Biochemistry, University of Verona, Verona, Italy; 8 UOSD SAFU, IRCCS-Regina Elena National Cancer Institute, Rome, Italy; 9 Department of Precision Medicine, University of Campania “L. Vanvitelli”, Naples and Biogem Scarl, Institute of Genetic Research, Laboratory of Precision and Molecular Oncology, Ariano Irpino, Italy

**Keywords:** Biomarkers, Single-cell, Functional omics, Tumor profiling, Personalized therapy

## Abstract

Molecular profiling of DNA and RNA has provided valuable new insights into the genetic basis of non-malignant and malignant disorders, as well as an increased understanding of basic mechanisms that regulate human disease. Recent technological advances have enabled the analyses of alterations in gene-based structure or function in a comprehensive, high-throughput fashion showing that each tumor type typically exhibits distinct constellations of genetic alterations targeting one or more key cellular pathways that regulate cell growth and proliferation, evasion of the immune system, and other aspects of cancer behavior. These advances have important implications for future research and clinical practice in areas as molecular diagnostics, the implementation of gene or pathway-directed targeted therapy, and the use of such information to drive drug discovery. The 1st international and 32nd Annual Conference of Italian Association of Cell Cultures (AICC) conference wanted to offer the opportunity to match technological solutions and clinical needs in the era of precision medicine.

## Presentation of the conference

The 1st international and 32nd Annual Conference of AICC was held at Magna Graecia University Campus S. Venuta, Catanzaro, on October 1st-2nd, 2019 with the Scientific Coordination of Dr. Maria Teresa Di Martino, from the same Academia. The conference was inaugurated by Prof. Michele Caraglia, President of the AICC. From circulating biomarkers, to mutational, transcriptomics and immunomics landscape, the meeting described a panorama of new platforms for personalization of therapy. This year conference also was an attempt to internationalize the traditional AICC annual conference, via the participation of internationally renewed Italian scientists and speakers coming from foreign countries, including the Nobel prize winner Bruce Alan Beutler, determining the success of the Conference. The organizers deeply thank those who took part in the conference and made it a success.

## Opening ceremony

The opening ceremony involved the academics authorities together with Prof. Beutler, Nobel Prize in Physiology or Medicine 2011, from University of Texas Southwestern Medical Center, Dallas (USA), who held a keynote lecture entitled “Inducing phenotypes, discovering them, and instantly solving them”. Prof. Beutler is the Director of the Center for the Genetics of Host Defense, and since its establishment Beutler’s team have produced nearly half a million of induced mouse germline mutations, covering almost all the models freely available for scientific use. Beutler is a pioneer in the study of innate immunity, and he was rewarded with the Nobel Prize for the discovery of the elusive sensing mechanism by which host cells recognize pathogens. Beutler spoke about the 25-years old challenge to find the receptor for lipopolysaccharide (LPS), also known as endotoxin, and about how he became interested in the question during the ‘80s, when he purified mouse tumor necrosis factor (TNF) and demonstrated that it was a key executor of LPS toxicity (systemic inflammation and death from septic shock). At that point, he began to wonder what the LPS receptor was, and therefore, how were microbes sensed by cells of the innate immune system. During his studies, Beutler used many methods in an attempt to find the LPS receptor, but ultimately only genetics led to a solution. By the use of C3H/HeJ and C57BL/10ScCr mice, carrying mutations of the LPS gene, in 1998 Beutler demonstrated that one of the mammalian Toll-like receptors, TLR4, acts as the membrane-spanning component of the mammalian LPS receptor complex [1]. In fact, he showed that destructive mutations of Tlr4 gene predispose to the development of Gram-negative sepsis, while leaving most aspects of immune function intact. The long path of the positional cloning research concluded with the discovery of TLRs won him the Nobel Prize in 2011.

While in the ‘90s it took him several years to track down a new gene, in the new millennium Beutler’s lab employed a new approach, based on “forward genetics”, to identify new genes involved in mammals’ immunity. In these studies, using the mutagen agent N-ethyl-N-nitrosourea (ENU), Beutler’s laboratory randomly generated a number of germline mutations in mouse models, detected about 200 mutations altering innate immune response and finally isolated them by positional cloning. Furthemore, by using the Linkage Explorer tool, Beutler’s team identifies the causative mutations among all candidate “phenotypic mutations” by browsing ENU-induced mutations and phenotypic effects. As multiple alleles of genes are acquired through mutagenesis, pooled “superpedigrees” are created to analyze the effects of mutations, automatically generated and analyzed by Linkage Analyzer [2]. This method has different advantages with respect to the conventional forward genetic methods, such as the unbiased declaration of mappable phenotypes, including those that are incompletely penetrant, the automated identification of causative mutations concurrent with phenotypic screening, the exclusion of genes not involved in phenotypes of interest. Notably, when phenotypic data are uploaded, the genetic cause of any phenovariance that may exist in the dataset is usually known within a few minutes.

Through the discovery of genes necessary for a particular process, Beutler’s lab continues gaining insight into the mechanism of a specific process and begins to understand the roles of individual genes. A full understanding of gene function forms the basis for developing effective treatments for human disease. Against this backdrop of growing knowledge, Beutler’s lab seeks to identify novel drugs and drug targets that may provide new strategies to treat diseases.

**Fig. 1:**
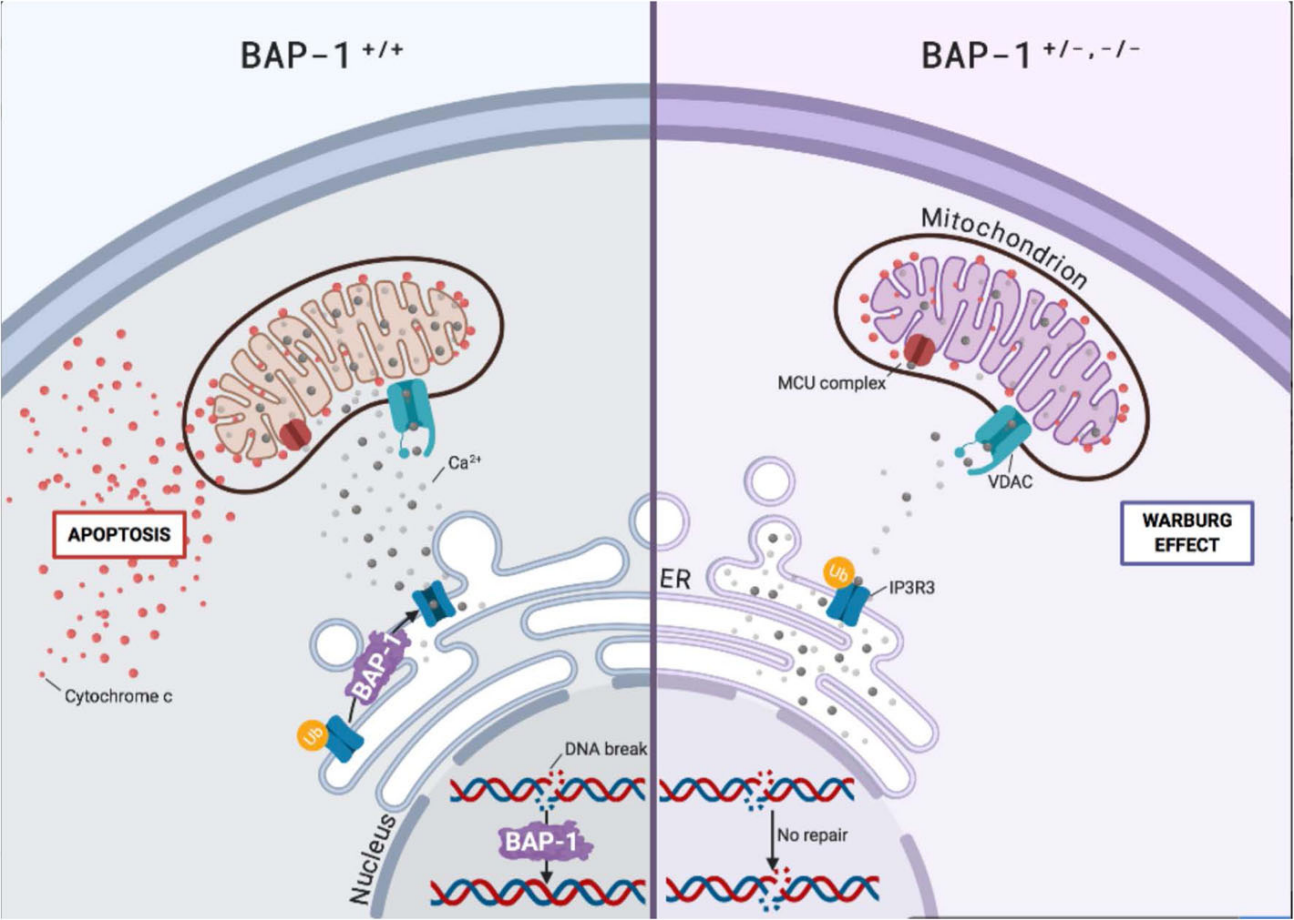
BAP-1 modulates DNA repair and Ca^++^ levels. Increased DNA damage is observed in BAP-1 mutant cells (BAP-1^+/−, −/−^) after exposure to asbestos, ultraviolet light, radiation, and chemotherapy as well as in cells with reduced BAP1 levels by siRNA experiments. In the nucleus, BAP-1 regulates DNA repair. In the cytoplasm, BAP-1 stabilizes by deubiquitylation (Ub) the IP3R3 receptor channel that in turn regulates Ca^++^ transfer from the endoplasmic reticulum (ER), in which Ca^++^ is normally stored, to the cytoplasm. Calcium ions, released from the ER, flow through the voltage-dependent anion channel (VDAC) located on the outer mitochondrial membrane, and then are actively transported inside the mitochondria by the mitochondrial uniporter channel complex (MCU) located on the inner mitochondrial membrane. Here, Ca^++^ is required for the normal activity of the Krebs’ cycle. In BAP-1^+/−, −/−^ cells, reduced Ca^++^ concentrations impair mitochondrial respiration (Krebs cycle), and the cells switch to aerobic glycolysis (Warburg effect). Moreover, in normal cells (BAP-1^+/+^), following a DNA damage that cannot be repaired, higher amounts of calcium ions are released from the ER and the consequently higher mitochondrial Ca^++^ concentration, causes the release of cytochrome c from mitochondria into the cytosol, thus starting the apoptotic cascade. In contrast, BAP-1 mutated cells, in which an insufficient amount of Ca^++^ is released, cannot start the apoptosis process, therefore the mutated cells are able to divide and may give rise to malignancy. Cartoon adapted from Carbone et al. [5]

Further along the conference, a distinguished lecture was held by Michele Carbone, from University of Hawaii, in Honolulu (USA), with the title “The BAP1 cancer syndrome: unique mechanisms and clinical characteristics requiring specific medical approaches”. This is a very exciting story running from approximately 25 years regarding the discovery of BAP1 mutations that define a novel cancer syndrome. Mesothelioma is caused predominantly by occupational or environmental exposure to asbestos and to other carcinogenic fibers and its incidence has decreased in Australia, USA, and Western Europe, where the use of asbestos has been rigorously regulated in the 1970s and 1980s, demonstrating the value of these preventive measures. In developing countries however, where large amounts of asbestos are still used, the overall mesothelioma mortality has not decreased; moreover, carcinogenic fibers are present in the environment and are disturbed as rural areas are being developed. Carbone, following his studies in Cappadocia, postulated that the epidemic mesothelioma observed in 3 villages of the area could not be attributed merely to erionite exposure, present in higher amounts in this environment, because only some families had mesothelioma cases in their pedigree despite sharing the same living environment. To shed light on this aspect, in 2001 Carbone’s team begun to study two US families affected by mesothelioma without a story of environmental exposure to carcinogenic fibers and demonstrated that all affected family members carried inherited germline mutations of the BAP1 gene. Germline BAP1 mutations-affected family members developed multiple malignancies, predominantly mesotheliomas and uveal melanomas, and less frequently skin melanomas and other tumors. Moreover, the discovery that individuals affected by the BAP1 cancer syndrome may develop benign melanocytic BAP1-mutated atypical intradermal tumors (MBAITs) provides an early marker to identify individuals at high risk of developing associated cancers [3]. In addition, Carbone’s group demonstrated that approximately 60% of mesotheliomas acquired BAP1 somatic mutation during tumor cell growth, underscoring the critical role of BAP1 in preventing mesothelioma growth. BAP1 is a deubiquitylase that modulates the activity of multiple genes and proteins involved in DNA replication, DNA repair, metabolism, and cell death, and has a strong tumor suppressor activity [4]. In particular, Carbone showed that BAP1 regulates both DNA repair and apoptosis as a result of DNA damage caused by asbestos, ultraviolet light, radiation, or chemotherapy. In fact, BAP-1 mutation inhibits asbestos-induced cell death, ensuring the viability of cancerogenic clones. In the cytoplasm, BAP1 modulates the stability of the IP3R3 channel, which allows the flux of Ca^2+^ from the endoplasmic reticulum into the mitochondria, where Ca^2+^ is required for the Krebs cycle and, at higher doses, promote cytochrome c release favouring apoptosis. As a consequence, reduced BAP1 levels in “normal” cells induce a Warburg effect, i.e., a shift from oxidative phosphorylation to aerobic glycolysis, a process that might prime these “normal” cells for malignant transformation and tumor growth. Therefore, high incidence of cancers in BAP1+/− carriers results from the combined reduced nuclear and cytoplasmic BAP1 activities (Fig. 1).

Current therapies extend the survival of mesothelioma patients only to ~ 1 year [6], whereas carriers of germline BAP1 mutations have an average survival of 5 years, an amount of time that significantly exceeded the benefit of any currently available therapy, thus suggesting that germline BAP1 mutations could be exploited as a prognostic factor [5, 7, 8]. The clinical translation of this discovery is the possibility to perform genotyping of patients and their relatives to identify carriers of germline mutations for screening programs and to conduct genomic studies on tumor biopsies to identify actionable mutations.

### SESSION I. Biomarkers for diagnosis, monitoring of progression and therapeutic response

Giuseppe Novelli from Tor Vergata University in Rome (Italy), held the first presentation of the session, focused on the use of biomarkers for the diagnosis, monitoring and therapeutic response of diseases. Non-communicable diseases (NCD) are chronic conditions such as diabetes, atherosclerosis, ischemia, heart attack, respiratory infections and tumors. Initially, a problem only for advanced countries, in recent years they have become among the main causes of death on the planet. These diseases are the result of complex interactions between economic growth, development, population aging, environmental changes and lifestyle.

To counteract the activation of mechanisms that can lead to the development of chronic diseases, it is essential to generate an innovative and personalized model of care. Translational genomics is the union of genetic research applied to the clinic passing through an in-depth analysis of results and digital innovation. Studies on the genetics of complex diseases pass through the improvement of available therapies avoiding individuals who could develop adverse events. To obtain reproducible results, it needs genetic studies that can identify which components of the measures are heritable and which are predictable need. Therefore, by studying and identifying genetic variants that influence pharmacokinetics, it is possible to formulate prognostic biomarkers able to identify the response to drugs in individuals, in order to carry out interventions and select therapeutic targets. Advances in whole-genome sequencing that identify genes involved in many diseases facilitate susceptibility to therapeutic treatment. Continuous identification of new genes and biomarkers specific to subtypes disease is essential for translation into personalized medicine [9]. The development of biomarkers to improve diagnosis, identify disease progression and patients who are more likely to respond to treatment, lead to a better risk stratification and facilitate decision-making in the clinical setting.

Overall, specific tumor mutations are identified followed by the synthesis of new antigens (peptides, miRNAs) or collections of platforms combined according to the individual’s tumor model [10]. Circulating miRNAs are assumed as excellent biomarkers for cancer as they regulate gene expression and are involved in cancer activation and development. To date the use of biological therapies for severe psoriasis can be effective for some patients but the therapeutic response can vary according to drug. The HLA-C*06:02 status has identified as a predictive biomarker that influence the response to most commonly used biologic treatments for psoriasis. The main reason to address personalized medicine approaches is to identify and treat only those patients who have a high potential to benefit from a particular therapy [11].

Currently only one biomarker is determined to guide the treatment, future molecular diagnostics may provide a simultaneous comprehensive profiling of multiple markers (multiplex testing). This means a movement from a single marker to a signature, and in the case of functional tumor profiling, this will allow us to select the most effective combination therapy for each patient.

Francesco Pepe from University Federico II of Naples (Italy), presented advances on evolution of liquid biopsy in oncology. During tumor progression, a strong selective pressure leads to the formation of resistant clones that proliferate rapidly and are less sensitive to drug treatment. To understand the molecular vision of solid tumors, biopsy samples are used, but unfortunately, sampling can often be damaged, limited in quantity and only partially highlights the heterogeneity of the entire tumor mass. Various studies have shown that the genetic profiles of tumor DNA obtained from cell-free DNA (cfDNA) and circulating tumor cells (CTC) strongly correspond to those of the tumor of origin [12]. This discovery has had important consequences both in molecular pathology and in clinical oncology.

Liquid biopsies are a multitude of minimally invasive techniques that can enable a real-time biomolecular characterization of the tumor through the analysis of human blood and other biological fluids such as urine, saliva, pleural effusions and cerebrospinal fluid [13]. In particular, circulating nucleic acids (ctDNA) are used to assess the response to a treatment that can normally induce drug resistance and even be able to assess the slightest residual disease [14].

In non-small cell lung cancer (NSCLC), very often, there is a poor availability of biopsies and cytological samples for immuno-histochemical and molecular analysis. This makes the use of liquid biopsies very attractive. For example, the analysis of the molecular state of the epidermal growth factor receptor (EGFR) by means of tumor ctDNA, extracted from plasma, is a valid alternative to tissues, in order to evaluate the suitability of patients for the administration of tyrosine kinase inhibitors (TKIs). The development of the next-generation sequencing method (NGS) allows the simultaneous evaluation of multiple somatic mutations. In addition to the hybrid capture technique, it is also possible to determine the presence of other genetic alterations, such as fusions. This approach achieved a marked increase in the detection of therapeutically targetable mutations. Pepe showed some results obtained by SiRe, a panel which covers 568 mutations in six genes (EGFR, KRAS, NRAS, BRAF, cKIT and PDGFRa) involved in NSCLC, gastrointestinal stromal tumor, colorectal carcinoma and melanoma [15]. It is interesting to note that out of a total of 322 NSCLC samples analyzed, only 28 (8.7%) failed to produce an adequate library, thus demonstrating the high performance of SiRe analytical parameters. Interestingly, the EGFR TKIs treatment based on SiRe genotyping lead to a clinical benefit in 82% of NSCLC patients thus highlighting the relevance of this tool for clinical application. Furthermore, recent advances in the technique of liquid biopsy have identified EML4-ALK translocation in the exosomal load (ExoALK) in NSCLC patients as a possible biomarker for the diagnosis and prognosis of this disease. The next perspectives will include early diagnosis and monitoring of the clinical benefit of drug treatment.

### SESSION II. Single cell profiling: from bench to bedside

The constant interaction between tumor microenvironment (TME) and infiltrating cells as a driving factor affecting tumor progression or immuno-surveillance was the fil rouge of the second session. Tumor evolution is indeed controlled by a fine balance between the decrease in effector cells (such as CD8^+^ T-lymphocytes, natural killer – NK – cells, M1-polarized tumor-associated macrophages, TAMs), endorsed by anti-tumor activity, and the increase in immuno-suppressive cells (such as T-regulatory – Treg - cells, myeloid-derived suppressor cells – MDSCs -, M2-polarized TAMs), characterized by a tumor-tolerant phenotype [16]. Specific oncogenic pathways activated in solid tumors, such as β-catenin pathway [17], often dictate the type and activity of immuno-infiltrating cells, tipping the balance between an effective control of tumor progression by the host immune system and a tumor-induced immuno-suppression. The tumor mutation burden is also critical: indeed it is well recognized in colon, breast and pancreatic ductal adenocarcinoma (PDAC) that higher is the mutation burden, higher is the spectrum of neoantigens generated and the possibility of inducing immuno-surveillance rather than immune-evasion [18]. Not only genetic alterations, but also epigenetic changes play a role in this balance.

In this second session, with an elegant series of experiments, Renato Ostuni, from San Raffaele Telethon Institute for Gene Therapy (SR-Tiget), Milano, Italy, unveiled an unexpected integration between inflammatory and immuno-suppressive stimuli on TAMs of PDAC. Indeed, in a complex inflammatory environment as TME, TAMs are exposed simultaneously to different cytokines, each activating completely different transcriptional and epigenetic programs. For instance, the concomitant exposure of macrophages to IL-4 and IFN-γ produced an antagonistic response, because IFN-γ inhibits the transcriptional and epigenetic programs induced by IL-4. Manipulating the ratio between the two cytokines reverses the resistance to IL-4, changing the macrophages activation [19]. In the context of PDAC, Ostuni groups’ highlighted that TAMs epigenetically up- or down-regulate the expression of inflammatory-related genes in response to different cytokine, alone or in combination. These changes linking the pro-inflammatory/immuno-suppressive signal of TME to the epigenetic reprogramming determine the fate of TAM in terms of polarization, pro-tumor or anti-tumor activities. Amazingly, the changes in epigenetic programs can be followed at single cell level. These observations indicate that TAMs are endorsed by a huge plasticity and heterogeneity [20], as already reported for other immuno-infiltrating populations such as neutrophils [21]. If such plasticity of TAMs and immuno-infiltrating cells justifies the high variability of responses to immuno-therapy, it should also be considered as the premise for a new treatment approach for PDAC, a tumor poorly responsive to chemotherapy and immuno-therapy. Indeed, a broad spectrum of immuno-modulators, ranging from cytokines to bacterial products such as LPS, may be used to reprogram TAMs at single cell level, forcing pro-tumor cells to become anti-tumor effectors. The work presented by Ostuni paves the way to a “precision immuno-therapy”, that could be a promising approach to counteract the progression of PDAC and of other cancers characterized by a strong immuno-evasive/immuno-suppressive phenotype.

Precision medicine is now a realistic vision in medical oncology but tumor cell heterogeneity represents a substantial hurdle, so the knowledge of the variations in tumor morphology, genetic or proteomic expression, might provide informations on the intra- and inter-tumor diversity and potentially on how the tumor cells might respond to treatment [22]. Tumor heterogeneity is frequently seen as the result of genetic evolution but additional mechanisms such as epigenetic modifications, transcriptional changes and alterations in protein levels or protein modifications in the tumor cells and/or the microenvironment also play a major role [23]. With the recent advances in technologies for single-cell analysis made during the past years, it is possible to monitor dynamic changes in tumor heterogeneity at genomic, transcriptomic, proteomic and functional levels.

**Fig. 2:**
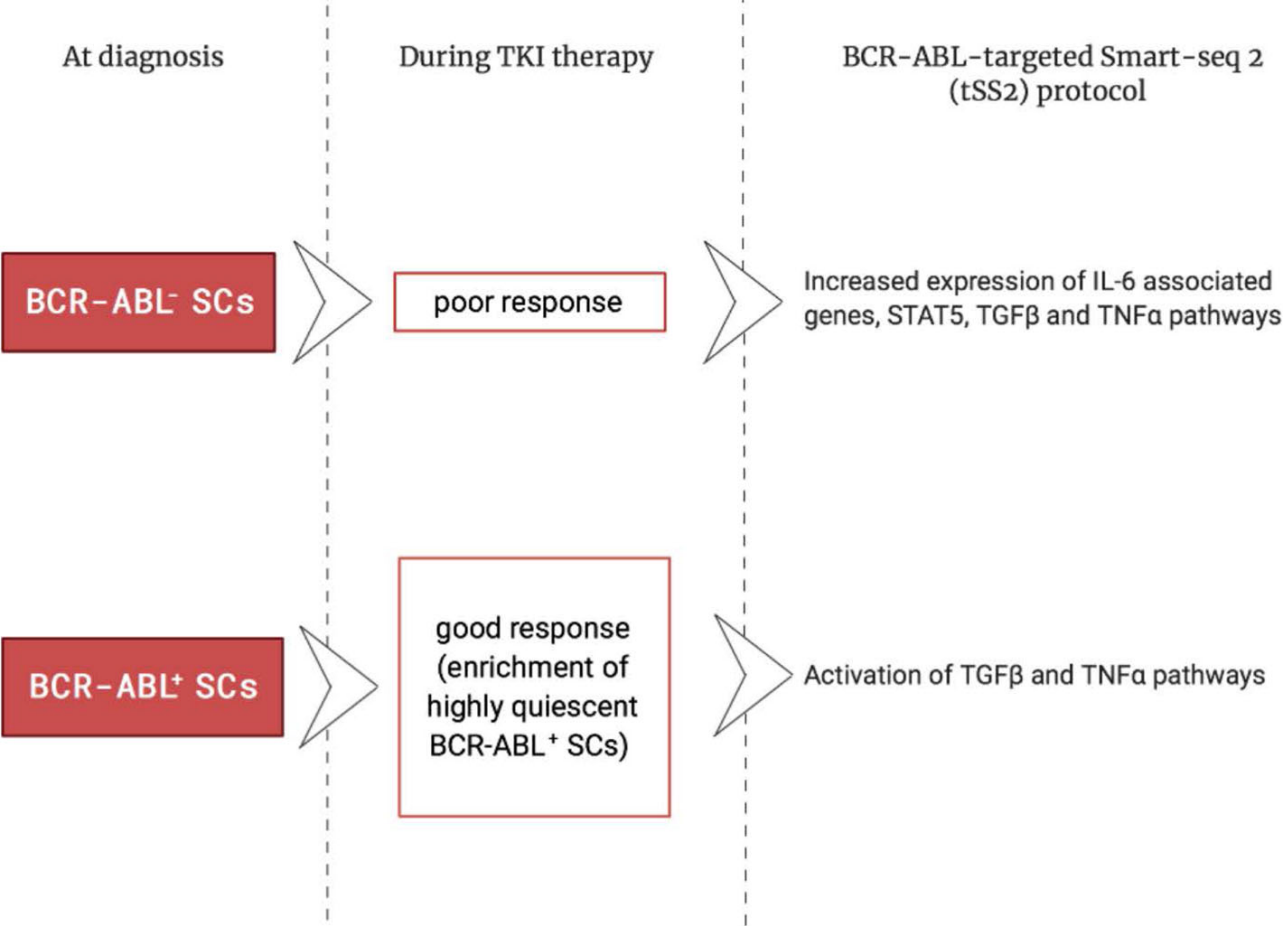
Single-cell transcriptomic of Chronic Myeloid Leukaemia Stem Cells (CML-SCs). The simultaneous single-cell mutation detection and RNA sequencing obtained by the novel BCR-ABL-targeted Smart-seq-2 protocol (BCR-ABL tSS2) permitted to study CML-SCs heterogeneity and to identify a subpopulation of resistant SCs, otherwise not possible through cell-population analysis. This approach allows to identify in resistant cells a number of dysregulation in genes and pathways which could be useful to predict disease response to therapy and targeted for precision medicine

In this context, Alice Giustacchini from Institute of Child Health in London (UK) reported a novel method to characterize cancer stem cells from Chronic Myeloid Leukemia (CML) patients who progressed after therapy with TKIs. CML is a clonal myeloproliferative disorder characterized by a chromosome translocation that generates the BCR-ABL oncogene encoding a constitutive kinase activity. Despite the high efficacy of TKIs which lead to the remission of disease, a high proportion of patients relapsed and this is the result of the propagation of CML stem cells (CML-SCs) which developed resistance to TKIs [24]. By combining single-cell RNA sequencing and high sensitivity targeted mutation detection, Giustacchini’s group developed a BCR-ABL-targeted Smart-seq-2 protocol (BCR-ABL tSS2) to study CML-SCs heterogeneity and to identify the signatures of these cells from diagnosis through remission post-TKIs administration and relapse. In particular, this strategy permitted to distinguish BCR-ABL^+^SCs and BCR-ABL^−^ SCs cells from normal hematopoietic stem cells (HSCs) and to analyze whole transcriptome with a highly specific and quantitative result in the single cell, at diagnosis, in the course of therapy with TKIs and at relapse with blast crisis progression (Fig. 2). Interestingly, they observed heterogeneity in BCR-ABL^−^ SCs with a distinct cluster already detected at diagnosis and the presence of this population was predictive of poor response as these patients later lacked to achieve major molecular response. An enriched expression of IL-6 associated genes and STAT5, accompanied by increased expression of genes involved in in TGF-β and TNF-α pathways, associated with increased SC quiescence, was reported in the BCR-ABL^−^ SCs and this observation correlates with the elevated serum levels of TNF-α and TGF-α in patient elicited poor response to treatment [25]. TGF-α and IL-6 plasma levels selectively identify CML patients who failed to achieve an early molecular response or progress in the first year of therapy. Moreover, Giustacchini reported the presence of rare persisting BCR-ABL^+^SCs in patient in the course of remission, already present at the diagnosis but enriched in highly quiescent cells. A transcriptionally pattern distinct from quiescent normal hematopoietic SCs was observed in BCR-ABL^+^SCs with activation of TGF-β and TNF-α pathways, representing possible targets for a new therapeutic combinatorial in CML. In summary, single-cell transcriptomic with high sensitivity detection of mutations provide insights into cellular and molecular mechanisms of resistance of CML-SCs to therapy and could be useful for predicting and monitoring therapy response and for the identification of new and more effective treatment to improve clinical outcome of this disease [26].

### SESSION III. Functional genomics in clinical practice

In recent years, the great progress in the development of methods for massive sequencing of large number of genomic sequences, termed NGS, has led to the accumulation of an incredible amount of data that can be used for a number of diagnostics, prognostic and therapeutic approaches, now approved for clinical purposes [27]. One of the main challenges in the use of such amount of data is the need to annotate genomic regions with their biological functions, associating molecular phenotypes to specific mutations in coding or non-coding sequences. This process is often classified as functional genomics and exploits different strategies based on genomic and transcriptomic protocols to identify new potential targets for applications in clinical practice.

In this context, Daniele Caracciolo from the Magna Graecia University, Catanzaro (Italy) reported data that highlight the relevance of genomic instability in multiple myeloma (MM), which is characterized by the presence of plasma cells harboring numerous and progressive karyotypic aberrations. Indeed, previous data have highlighted mutation or deletion of genes implicated in DNA damage response in 22% of MM patients [28]. Further investigations by Caracciolo and coworkers have provided evidence that increased expression of the DNA ligase III (LIG3) correlates with worst prognosis in MM and is crucial for genomic instability and survival of multiple myeloma cells. LIG3 plays a fundamental role in Alternative Non Homologous End Joining (Alt-NHEJ) repair, which may be a cause for genomic instability. Indeed, increased LIG3 mRNA expression in multiple myeloma patients correlates with shorter survival and with more advanced stage of disease [29].

Interestingly, experiments performed by Caracciolo and coworkers have shown that knockdown of LIG3 reduces MM cells viability, suggesting that neoplastic plasma cells are dependent on LIG3-driven repair. This observation has prompted the search for new potential LIG3 inhibitors, useful for clinical application, which is still in progress. Investigation of the post-transcriptional mechanism of regulation of LIG3, has led to the identification of miR-22-3p as a negative regulator of LIG3. Enforced expression of miR-22 in MM cells effectively downregulated LIG3 protein, which in turn increased DNA damage, to a level that inhibited in vitro and in vivo cell growth, inducing apoptotic cell death [29]. Further unpublished data by Caracciolo and coworkers show the involvement of Myc in the transcriptional regulation of Lig3, miR-22 and PARP1, prompting the identification of another mechanism through which Myc plays a role in MM tumorigenesis [Unpublished data].

Giovanni Tonon, from the Center for Translational Genomics and Bioinformatics, San Raffaele Scientific Institute, Milan (Italy), again digging from data obtained in MM genome sequencing, focused on DIS3, a 3′-5′ exoribonuclease which is mutated in up to 18% of MM patients. DIS3 is the catalytic core of the RNA exosome, involved in turnover of normal mRNAs, maturation of precursor RNAs and RNA quality control (degradation of aberrant RNA precursors, control of mRNA expression levels, degradation of promoter upstream transcripts). Tonon demonstrates that DIS3 depleted cells show widespread increase of DNA:RNA hybrids, ending in an increased genomic instability, thus suggesting that DIS3 is also engaged in DNA damage response, in particular in the NHEJ repair. Indeed, loss of DIS3 increases DNA:RNA hybrids and impairs DSB repair, inducing a BRCAness status and accumulation of mutations. MM samples analysis has confirmed that DIS3 mutated tumors present higher mutational burden, associated with an interferon I response [30]. Furthermore, Tonon has provided an essential contribution to the topic of tumor resistance to treatments: tumors cells may exhibit a reversible drug-tolerance, induced by epigenetic changes in phenotypically distinct subpopulations of cells, which allows survival of the tumor and further tumor evolution and progression after the initial elimination of the tumor bulk and suspension of the treatment. This phenomenon highlights the need for single cell analysis and study of single cell cancer evolution in the clinic. Dissection of the modifications undergone by tumor cells after the initial drug treatment allows the identification of different pattern of expressions and genomic alterations, that may be due to the formation of extrachromosomal circular DNA (eccDNA), double minutes, which provides a different genotype/phenotype, favouring cancer evolution.

Andrea Mafficini, from the ARC-Net Centre for applied research on cancer and the Department of Diagnostics and Public Health, University and Hospital Trust of Verona (Italy), has presented data on the genomic landscape of PDAC. These tumors include well-differentiated Pancreatic Neuroendocrine Tumors (PanNETs), featuring a variable prognosis from indolent to aggressive, and poorly differentiated Pancreatic Neuroendocrine carcinomas (PanNEC), which show a worst prognosis like that of PDAC. PanNETs and PanNEC differ also in term of genetic alterations being PanNEC genetically characterized by TP53 and RB1 inactivation while PanNETs are more heterogeneous. Mafficini underlined that the current challenge is to identify signature for actionable pathways for these rare tumors. Mafficini summarized several sets of potential targets, including Wnt pathway, cell cycle regulation, PI3K/mTOR, Chromatin remodeling, homologous recombination and DNA single base repairs [31]. Mafficini also illustrated the Master registry trial in which samples from patients with rare tumors without curative options were explored by whole genome sequencing and RNAseq. Based on molecular profiling and clinical interpretation of molecular data by a molecular tumor board, were suggested indications of genomics-guided treatment, with evidences of relevant improvement of response in several cases. The possibility that a comprehensive gene panel may be a better choice for the identification of the genes that matter the most. Unfortunately, most commercial and custom cancer panels show poor concordance in gene selection, likely due to lack of objective methodologies, and most panels lack of adequate coverage of structural variants, which are the majority of clinically informative data. To this end, the International Cancer Genome Consortium has the goal to obtain a comprehensive description of genomic, transcriptomic and epigenomic changes in 50 different tumor types or subtypes [32]. Another issue highlighted by ultradeep sequencing is the intratumor heterogeneity, and the presence of low abundance mutations which may confer clonal resistance to therapy. These large-scale data allowed to place previous findings in a more defined frame and provided potential markers for patient stratification and companion diagnostics.

Finally in this session, Javier De Las Rivas, from the Grupo de Investigacion en Bioinformatica y Genomica Funcional Cancer Research Center (CiC-IBMCC, CSIC/USAL/IBSAL), Consejo Superior de Investigaciones Científicas and University of Salamanca, Salamanca (Spain), talked about the analysis of cancer genomic data in order to achieve better patient stratification and personalized molecular profiling. Javier De Las Rivas underlined that cancer is not a genetic disease affecting only one or few genes, but instead it is a “genomic disease” in which many genes are involved. Moreover, differences in the panel of genes involved are not only from different type of cancer, but also in each patient and at different stages of the disease. Indeed, De Las Rivas discussed the great difficulty to know in the processes of identifying biomarkers for specific individuals or subtype of tumours. The application of omic technologies in cancer research lead to the generation of thousands of data that can only be useful if advanced computational and statistical methods are available for their analysis. De Las Rivas’ group developed this type of methods, including the DECO method (decomposing heterogeneous cohorts using omics data profiling) [33]. This new method allows to find significant association between biological features (biomarkers) and samples in large scale omics data. DECO greatly enhances the discovery of biomarkers, and the accurate patient stratification. They also described a robust strategy to combine transcriptomic expression data with patient survival data. The appropriate integration of omic and survival clinical data is a necessary step to build risk predictors and apply these predictors to the prognosis of patients.

At the end of this session a distinguished lecture was held by Gerry Melino, from Medical Research Council, Toxicology Unit, Cambridge (UK), with title “ZNF281 contributes to the DNA damage response and is a prognostic maker for neuroblastoma”. He illustrated the recent results obtained in his lab supporting the opportunities for cancer patients’ stratification and for the development of personalized therapeutic strategies. ZNF281 is a zinc-finger factor affecting cell death, which expression increases after genotoxic stress caused by DNA-damaging drugs. ZNF281 silencing significantly increases cell death upon chemotherapy in vitro by regulating XRCC2 and XRCC4, two genes that take part in homologous recombination and non-homologous end joining, respectively [34]. Both are transcriptionally activated by ZNF281 through a DNA-binding-dependent mechanism, involving an interaction with c-Myc. Conversely, ZNF281 decreases during neuronal differentiation of embryonic stem cells and in retinoic acid-induced differentiation of neuroblastoma (NB) cells. Silencing of ZNF281 induces neuronal differentiation of NB cells while its ectopic expression causes the opposite effect. Furthermore, the expression of ZNF281 is inhibited by TAp73 through miR-34a. Contrariwise, MYCN promotes the expression of ZNF281 at least in part by inhibiting the expression of miR-34a. These findings imply a functional network that includes p73, MYCN and ZNF281 in NB cells where ZNF281 acts by inhibiting neuronal differentiation. Array analysis of NB cells silenced for ZNF281 expression identified GDNF and NRP2 as two transcriptional targets inhibited by ZNF281. Bioinformatic analysis of NB datasets indicates that ZNF281 is a prognostic marker of aggressive, undifferentiated NB. These observations suggest that ZNF281 is a regulator of cell death during DNA Damage Response as well as of neuronal differentiation, with relevant implication in cancer. Finally, Melino’s group identified a novel role for the zinc-finger protein ZNF281 in regulating the ordered recruitment of the NHEJ repair factor, XRCC4, at damage sites. ZNF281 is recruited to DNA lesions within seconds after DNA damage through a mechanism dependent on poly-ADP ribose polymerase (PARP) activity and on its DNA binding domain [35]. ZNF281 binds XRCC4 through its zinc-finger domain and facilitates its recruitment to damaged sites. Consequently, depletion of ZNF281 impairs the efficiency of the NHEJ repair pathway and decreases cell viability upon DNA damage. Survival analyses from datasets of commonly occurring human cancers, show that high levels of ZNF281 correlate with poor prognosis of patients treated with DNA-damaging therapies [36]. In conclusion Melino’s lab results define a late ZNF281-dependent regulatory step of NHEJ complex assembly at DNA lesions supporting its additional role as marker for cancer patients’ stratification for therapeutic approaches in NB treatment.

### SESSION IV. Functional immunomics in precision medicine

The recent clinical successes of immune checkpoint blockade and chimeric antigen receptor T (CAR-T) cell therapies represent a turning point in cancer immune-therapy and underscore the importance of understanding basic tumor immunology for successful clinical translation in treating patients with cancer [37], also recognized by Nobel Prize awarded in the year 2018 to immunologist scientist [38].

This session was dedicated to the usage of immunomics as a new research tool in systems biology of cellular immune responses in order to ameliorate cancer immuno-therapy. Currently, cancer genome sequencing and cancer proteomics are useful for diagnosis and personalized treatment. Analysis of these omics data involves integration of computation, statistics and systems biology methods. The amalgam of such methods, which help study interaction of cancer cells with TME, harnessing immune system for cancer therapy or its prevention through vaccines, has led to the foundation of immune-related genomics and proteomics, namely cancer immunomics [39]. However, we are still far away from making precise, quick and reliable diagnostic and treatment predictions. We need decision support systems to facilitate diagnosis, tumor evaluation prediction and assessment of individual profile for making functional immunomics a personalized clinical reality. Pierpaolo Correale, director of the oncology unit of the hospital of Reggio Calabria (Italy), discussed new improvements in inflammosomics in the prediction of response to immuno-therapy. In particular, he reported the results of multiple studies suggesting that that baseline inflammatory status, occurrence of severe immune-related adverse events (irAEs) and rise in serum auto-antibodies (AABs), as ANA, ENA, and ANCA may be a reliable early predictor of response in metastatic NSCLC patients treated with nivolumab, a human recombinant IgG-1 addressed to impair the PD-1/PDL-1 immune-checkpoint [40]. Moreover, Luigi Buonaguro from IRCSS Fondazione Pascale in Naples (Italy), discussed on the necessity to identify effective antigenic targets for cancer immuno-therapy, especially in hepatocellular carcinoma (HCC). Indeed, the majority of HCC patients can be treated only with non-surgical loco-regional therapies with extremely variable survival rates and tumor recurrence in 50 to 80% of patients at 5 years after treatment. The small kinase inhibitor sorafenib is, together with the anti-PD1 antibody Pembrolizumab, one of the few drugs approved for the systemic treatment of advanced unresectable HCC providing a limited increase in survival. In this framework, a therapeutic cancer vaccine may represent an effective strategy to cure HCC. Notably, unlike several other cancers, only few cancer vaccine trials for HCC have been conducted so far with yet modest results, indicating that improvements in several aspects need to be implemented. In particular, identification of novel specific tumor antigens, evaluation of delivery systems and combinatorial strategies to counteract the immuno-suppressive microenvironment, may result in unprecedented clinical outcomes with great beneficial effect for HCC patients. Overall, new technical advancements will foster development of ready-to-use chips for easy and rapid screening of vaccines to improve the outcome of vaccinations. Systems vaccinology, that is the global approach applied to vaccinology from discovery of new antigens to evaluation of vaccine efficacy, represents the real turning point for the switch from the “empirical” to the “knowledge-based” age of the vaccinology, enabling the development of even more successful vaccines for preventive as well as therapeutic intervention strategies for cancer immunotherapy [41]. Finally, Sabrina Arena from University of Turin and Candiolo Cancer Institute, Turin (Italy), discussed the clonal cancer evolution as therapeutic target. In particular, the response of colorectal cancer (CRC) to monoclonal antibodies against EGFR, such as cetuximab or panitumumab, is often transitory and it is due to the progressive emergence of acquired resistance. By using a liquid biopsy approach that allows the detection of emerging resistant cancer clones before relapses are clinically manifest, they discovered that a multistep clonal evolution of drug resistant cells underlies the development of resistance to anti-EGFR antibodies in cells and CRC patients. Intriguingly, this supports the general concept that CRC cells likewise exploit adaptive mutability to evade therapeutic pressure [42]. She further reported that the generation of new mutations is accelerated when genes involved in DNA repair pathways are altered and that the manipulation of the mutational burden can trigger persistent therapeutic responses. It emerged that the rational combination of targeted- and immuno-therapies may restrain tumor evolution and limit the emergence of drug resistance thus leading to long-term effective responses.

### Closing lecture and remarks

In the closing lecture of the meeting, “Driving the CAR beyond the checkpoint”, Francesco Marincola from Refuge Biotechnology, Menlo Park (USA), has presented the most important features of the evolution of anti-cancer immunological therapy in the direction of superpersonalized anti-cancer therapy with CAR-T. It is an established knowledge that cancer cells/tissues have a constitutive resistance to immunological effectors that allows them to establish and grow in the host disseminating in distant organs. Observations in humans based on transcriptional profiling converge into what we call an ‘immunologic constant of rejection’ that characterizes some diseases from organ rejection to autoimmunity and cancer. This constant includes the coordinate activation of interferon-stimulated genes and immune effector functions. Understanding this final effector pathway may suggest novel strategies for the induction or inhibition of tissue-specific destruction with therapeutic intent in cancer in order to overcome immunological escape [43]. On this light, recent studies have found that Interferon gamma-related profile predicts clinical response to immunological check point inhibitors [44]. In fact, responses are dramatic and long lasting but occur in a subset of tumors and are largely dependent upon the pre-existing immune contexture of individual cancers. Available data suggest that three landscapes best define the TME: immuno-active, immuno-deserted and immuno-excluded. This trichotomy is observable across most solid tumors (although the frequency of each landscape varies depending on tumor tissue of origin) and is associated with cancer prognosis and response to checkpoint inhibitor therapy (CIT). Various gene signatures (e.g. Immunological Constant of Rejection and Tumor Inflammation Signature) that delineate these landscapes have been described. In an effort to explain the mechanisms of cancer immune responsiveness or resistance to CIT, several models have been proposed that are loosely associated with the three landscapes [45]. One of these models proposes the existence of mechanical (physical stop of the contact between cancer and T cells), functional (biological or metabolic interactions between tumor microenvironment cells and immune cells limiting migration, function and/or survival of T cells) and dynamic barriers (T cell/ cancer cell interaction that limit the anti-cancer functions of T cells such as immunological check points) that hamper the anti-cancer effects of immune effectors in the tumour tissues. In order to overcome these resistance mechanisms, CAR-T cells could represent a good opportunity. Human T cells engineered to express a chimeric antigen receptor (CAR) specific for folate receptor-α (FRα) have shown robust antitumor activity against epithelial cancers in vitro but not in the clinic because of their inability to persist and home to tumor in vivo. Therefore, CARs were constructed containing a FRα-specific scFv (MOv19) coupled to the T-cell receptor CD3ζ chain signaling module alone (MOv19-ζ) or in combination with the CD137 (4-1BB) costimulatory motif in tandem (MOv19-BBζ). Transfer of human T cells expressing the costimulated MOv19-BBζ CAR mediated tumor regression in immuno-deficient mice bearing large, established FRα(+) human cancer. MOv19-BBζ CAR T-cell infusion mediated tumor regression in models of metastatic intraperitoneal, subcutaneous, and lung-involved human ovarian cancer. Importantly, tumor response was associated with the selective survival and tumor localization of human T cells in vivo [46]. In conclusion, the paradox of immune exclusion could be reverted using superpersonalized immunological therapy based upon gene editing of transplanted immunological effectors.

## Conclusion

In conclusion of the 1st international and 32nd Annual Conference of Italian Association of Cell Cultures (AICC), it is important to highlight that, based upon the massive data and results derived from the comprehensive study of the molecular (both protein and genetic) profile of human tumours and of the different cellular compartments of the cancer tissues, a new scenario of intervention and management of the cancer disease is emerging. The use of deep sequencing and proteomic technologies in the study of cancers is also becoming attractive and essential in the diagnostic workflow of cancer patients and the personalization of therapy, in parallel with the discovery of new targets both in cancer and micro-environmental cell components. The organizing committee hopes that this message reached the over 200 participants, most of them young researcher, coming from the most important research and clinical Italian institutions.

As for more than 30 years now, during the meeting young researchers were awarded for either best posters or oral presentation and for that we thank the Molecular and Translational Oncology PhD Program of Magna Græcia University of Catanzaro and the Journal of Experimental and Clinical Cancer Research in supporting AICC conference awards.

Finally, we thank all the participant who guaranteed success to the AICC Annual Conference.

## Data Availability

Not applicable.

## Abbreviations

AICC: Italian association of cell cultures
Alt-NHEJ: Alternative-Non homologous end joining
BCR-ABL tSS2: BCR-ABL-targeted Smart-seq-2
cfDNA: Cell-free DNA
CIT: Checkpoint inhibitor therapy
CML: Chronic myeloid leukemia
CML-SCs: CML stem cells
CRC: Colorectal cancer
CTC: Circulating tumor cells
eccDNA: Extrachromosomal circular DNA
EGFR: Epidermal growth factor receptor
ENU: N-ethyl-N-nitrosourea
HCC: Hepatocellular carcinoma
HSCs: Hematopoietic stem cells
irAEs: Immuno-related adverse events
LIG3: Ligase III
LPS: Lipopolysaccharide
MBAITs: Mutated atypical intradermal tumors
MM: Multiple myeloma
NB: Neuroblastoma
NCD: Non-communicable diseases
NGS: Next-generation sequencing
NSCLC: Non-small cell lung cancer
PanNEC: Pancreatic neuroendocrine carcinomas
PanNETs: Pancreatic neuroendocrine tumors
PARP: Poly-ADP ribose polymerase
PDAC: Pancreatic ductal adenocarcinoma
TAMs: Tumor-associated macrophages
TKIs: Tyrosine kinase inhibitors
TLR: Toll-like receptor
TME: Tumor microenvironment
TNF: Tumor necrosis factor
